# EDGAR: A software framework for the comparative analysis of prokaryotic genomes

**DOI:** 10.1186/1471-2105-10-154

**Published:** 2009-05-20

**Authors:** Jochen Blom, Stefan P Albaum, Daniel Doppmeier, Alfred Pühler, Frank-Jörg Vorhölter, Martha Zakrzewski, Alexander Goesmann

**Affiliations:** 1 Computational Genomics, Center for Biotechnology (CeBiTec), Bielefeld University, Bielefeld, Germany; 2 Bioinformatics Resource Facility, CeBiTec, Bielefeld University, Bielefeld, Germany; 3 Institute for Genome Research and Systems Biology, CeBiTec, Bielefeld University, Bielefeld, Germany

## Abstract

**Background:**

The introduction of next generation sequencing approaches has caused a rapid increase in the number of completely sequenced genomes. As one result of this development, it is now feasible to analyze large groups of related genomes in a comparative approach. A main task in comparative genomics is the identification of orthologous genes in different genomes and the classification of genes as core genes or singletons.

**Results:**

To support these studies EDGAR – "Efficient Database framework for comparative Genome Analyses using BLAST score Ratios" – was developed. EDGAR is designed to automatically perform genome comparisons in a high throughput approach. Comparative analyses for 582 genomes across 75 genus groups taken from the NCBI genomes database were conducted with the software and the results were integrated into an underlying database. To demonstrate a specific application case, we analyzed ten genomes of the bacterial genus *Xanthomonas*, for which phylogenetic studies were awkward due to divergent taxonomic systems. The resultant phylogeny EDGAR provided was consistent with outcomes from traditional approaches performed recently and moreover, it was possible to root each strain with unprecedented accuracy.

**Conclusion:**

EDGAR provides novel analysis features and significantly simplifies the comparative analysis of related genomes. The software supports a quick survey of evolutionary relationships and simplifies the process of obtaining new biological insights into the differential gene content of kindred genomes. Visualization features, like synteny plots or Venn diagrams, are offered to the scientific community through a web-based and therefore platform independent user interface , where the precomputed data sets can be browsed.

## Background

The mid fifties produced a rather pragmatic definition of the term species, described as a group of cultures or strains which is accepted by bacteriologists as sufficiently closely related [1]. About thirty years later a more fundamental proposition of the term [2] considered measurable quantities including strains' DNA molecules reassociation values and phenotypic traits. However, in recent times, these classical approaches are likely to be outdated by future deductions which may be taken from the increasing collection of genomic information.

Especially methods of pyrosequencing have the undisputed potential to yield huge amounts of genomic sequence information in relatively short time spans. Unsurprisingly, the number of complete genomes being published is rapidly increasing (see ).

As a consequence, one may ask if the genetic variability of a species can be described using only one single strain. A closer look at numerous pieces of circumstantial evidence apparently negates this question, such as the comparison of the well known *Escherichia coli *strain K12 and its relative O157:H7 revealed 1387 genes to be specific to a certain strain, or the comparison of 17 *Streptococcus pneumoniae *strains by Hiller *et al*., where the clustering of similar genes revealed clusters exclusive to one or some strains. Interestingly even isolates taken at nearby locations from patients with similar symptoms showed divergent genotypes [3].

Inspired by the Greek word "pan" for "whole", Tettelin *et al*. shaped the idea of the pan genome [4]. Using whole-genome shotgun sequencing, they gained genomic information of six strains of *Streptococcus agalactiae*. In comparison with two additional publicly available genomes of the major pathogenic serotype of *Streptococcus agalactiae*, called group B *Streptococcus *(GBS), they found a significant amount of genes not being shared among the compared strains. Their discoveries led to the definition of the pan-genome constituting that "a bacterial species can be described by its pan-genome, which is composed of a 'core genome' [⋯] and a 'dispensable genome' ".

Furthermore, a differentiation between open and closed pan-genomes has been introduced by Medini *et al*. [5]. While for example the genomes of *Buchnera aphidicola *showed almost no gene rearrangements (lateral exchange) and therefore its pan-genome is denominated as closed [6], the compared strains of *Streptococcus agalactiae *form an open pan-genome, i.e. every newly sequenced strain would contribute new genes into the pool of available genes for that specific species.

Muzzi, Masignani, and Rappuoli pointed out the importance of these concepts, not only to study genetic diversity, but also in terms of medical discoveries and cures [7]. During the design and analysis of potential vaccines, where methods like the reverse vaccinology approach play an important role, the genes of the core genome are most likely the most desirable targets for novel drug candidates.

An automated calculation of the characteristics of a species's pan-genome is highly desirable to identify singleton genes, the dispensable and the core genome. Different tools have been developed to compare the sequences of genomes, comprising for example the VISTA family of tools, xBASE or GeConT [8-11]. However, when these tools were designed, attention was focused on the comparison of the genomes of different species. In the mean time, particularly resulting from the upcoming pyrosequencing technologies, bioinformatics support for the comparison of multiple strains of the same species was needed. Therefore databases like the Comprehensive Microbial Resource (CMR) or the Microbial Genome Database (MBGD) were designed dedicated to the comparison of multiple genomes of related species [12,13].

The CMR provides numerous comparative tools for analysis of 438 genomes stored in its database, including a multi-genome homology comparison tool. This tool allows the user to calculate the number of proteins in a reference genome that have hits to up to 15 selected comparison genomes. The resulting homologous genes of the selected genomes are presented in a circular display of proteins the selected genomes have in common. Special sets of these homologous genes like the core genes or the singletons can be observed and exported in a tabular format. The MBGD provides comparative analysis features for 631 finished bacterial genomes. The genes of selected genomes can be clustered to homologous groups, resulting in a set of ortholog clusters. From this ortholog clusters the core genome, the pan genome and the singletons of a given genome can be calculated. Additional analysis and visualization features are available for the clustered genes like multiple alignments or a comparison of the context of the genes on a genome map. While both databases provide a wide range of highly valuable analysis features, both have limitations in the analysis of groups of related genomes. When using the CMR the user can only view the core genes and singletons for the reference genome. Homologous protein mappings can be analyzed only for the comparison of the reference genome to one other genome, an overall table for all genomes is missing. Additionally, the pan genome can't be displayed using the CMR. The MBGD can calculate the core genome as well as the pan genome or singleton genes, but it is focused on the calculated ortholog clusters. There is a lack of genome wide analysis features like Venn diagrams of the common gene pools of the analyzed genomes or synteny/scatter plots of homologous proteins, and the web interface is not very intuitive. Furthermore, both databases don't feature phylogenetic analyses.

Another crucial aspect when analysing groups of related genomes is the definition of a homology criterion to cluster genes together. Both databases offer a selection of parameters to the user to define a homology cutoff for the genome comparisons. When using the MBGD one can choose amongst 16 parameters in different combinations. The CMR offers three parameters: Minimum percent similarity, minimum percent identity, or maximum p-value. A user can use the default parameters or has to find the parameters best suited for the genomes he wants to compare by trial and error. An automatic estimation of an adequate homology criterion would be a great easement of comparative analyses. Therefore we developed EDGAR as an easy-to-use integrated solution, capable of performing genome comparisons and phylogenetic analyses of multiple strains of a species based on a homology cutoff automatically adjusted to the analyzed genomes.

## Implementation

EDGAR is a bioinformatics approach to provide quick access to orthology information and comparative genomics.

### System design

The system design of EDGAR is based on a standard three-tiered architecture. As a data backend (database layer), we use SQLite . SQLite is an easy-to-use file based relational database management system and allows simple transfer of complete data sets from one operating or hardware system to another one. The business logic layer is implemented in Perl  using the DBI package to access the data backend. We created a user interface (presentation layer) based on Perl CGI and some JavaScript. The setup of an EDGAR project requires the selection of related genomes. FASTA files for all coding sequences of every genome and their corresponding NCBI protein table files (.ptt), and BLAST databases need to be stored in a project directory. For the precalculated projects data from the NCBI genomes database  was used. Optionally, an EDGAR project can be set up based on an existing project of the automatic annotation plattform GenDB [14], comparing all or a selection of genomes in the project.

We have implemented several maintenance scripts to set up a project and perform all required computations like the creation of phylogenetic trees. All calculations are realized using object oriented Perl. The all-against-all comparisons of the genes of a genus group are distributed over a compute cluster using Sun Grid Engine .

### BLAST score ratio values

As described in the background section the definition of an adequate orthology criterion is a task of vital importance. Following the original definition of orthology by Fitch [15], two genes are orthologs if they diverged through a speciation event. But due to the fact that orthologs are mainly used to propagate functional annotations, the term "ortholog" is often used to describe genes with conserved function. The majority of scientists uses bidirectional best hits (BBHs) of the well known alignment tool BLAST [16] and chooses a certain e-value or an identity threshold over a given alignment length to define orthologous genes. Various more sophisticated orthology identification approaches have been developed in the past, e.g. Clusters of Orthologous Genes (COG), InParanoid, OrthoMCL, Ensembl Compara, Homologene, RoundUp, EggNOG, or OMA [17-24]. Some of these approaches were benchmarked by Hulsen *et al*. with the result that, while InParanoid performs best, BBHs can give a good orthology estimation for closer related species [25]. Recently, Altenhoff *et al*. confirmed the good performance of BBHs in a comparison of 11 orthology estimation methods and concluded that BBHs give comparable results to the more sophisticated methods for both the "phylogenetic" orthology definition by Fitch and the widespread "functional" definition [26]. A drawback of BBHs is that only one-to-one orthologous pairs are found, for duplicated genes or paralogs only a single hit will be found, the following ones will be missed. But as the BBH calculation is straightforward and therefore fast enough to handle the huge amounts of sequence information in comparative genomics, this drawback has to be accepted and we use BBHs as orthology criterion in EDGAR. For all calculations protein BLAST (blastp) was used with BLOSUM62 as similarity matrix.

**Figure 1:**
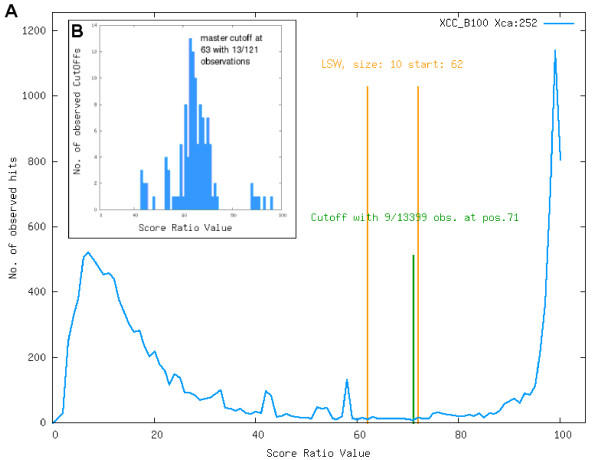
**BLAST score ratios**. (A) Histogram of the SRVs (multiplied by 100 to gain percent values) resulting from the comparison of Xcc B100 and Xca 756C. The distribution of the SRVs is clearly bimodal with one peak at 7% and one peak at 98%. The *lowest scoring window *(LSW) with a size of 10 was estimated at positions 62 – 72, the lowest single value at position 71, thus giving a cutoff of 71% for this genome comparison. The histogram of the calculated cutoffs for all 121 possible comparisons of *Xanthomonas *genomes can be seen in panel (B). The calculated cutoffs show a normal distribution with a peak 63%, by this defining the master-cutoff for the orthology estimation among *Xanthomonas *genomes.

For the completely automated calculation of genome comparisons it is crucial to rely on a generic orthology criterion consistent within the genome group. For this reason orthology thresholds were generated based on so called BLAST Score Ratio Values (SRVs) (as suggested by Lerat *et al*. [27]) for every compared genus. Instead of using the absolute scores of every BLAST hit, this method employs the BLAST bit scores in relation to the maximum bit score. While Lerat *et al*. used a fix SRV threshold of 30 for orthology estimation, we use a sliding window approach to estimate the appropriate cutoff for every genus. The maximum score is defined as the score resulting from an alignment of a gene against itself. As a BLAST self hit always has 100% identity over 100% of the query sequence length one gains the maximum bit score possible for the query gene. All hits of a query gene versus genes from other genomes are normalized regarding to this maximum score, so the SRV is defined as the ratio (*Observed score/Maximum score*), thus giving a value in the range [0, 1]. The SRVs for all BLAST hits of a genome versus another are plotted as a histogram, in most cases forming a bimodal distribution (see Figure 1). The first peak at low similarity values probably represents nonspecific hits, while the second peak represents orthologous genes. To remove all nonspecific hits, a cutoff value has to be identified that discriminates between the two peaks automatically. To find this cutoff, we used a sliding window of a given width over the histogram, thus searching for the lowest scoring window (LSW), which is the window with the lowest count of BLAST hits. Within this LSW, the SRV value with the lowest hit count is estimated, yielding the final cutoff value. A SRV distribution with the search window and the resulting cutoff is shown in Figure 1A. This procedure is repeated for all possible combinations of genomes, resulting in *n*^2 ^combinations for a number of *n *genomes. The *n*^2 ^cutoffs are plotted in a second histogram, in most cases showing a normal distribution (see Figure 1B). To get a comparable general threshold for all subsequent calculations, the peak of this histogram is determined and used as so called master-cutoff.

The orthology cutoff generated by this approach is quite strict, as all low quality BLAST hits are filtered out. For the Xanthomonas project with a calculated master cutoff of 63 this results in only 44% of all BLAST hits passing the filter. The minimum percent identity of BBHs passing the filter is 53.75% and mean BLAST e-value is lower than 1*e*^-10^. As a consequence of that strict threshold orthologs found by EDGAR, especially when conserved among numerous genomes like the core genes, could be considered real orthologs, but some potential orthologs might be lost.

#### Limitations

**Figure 2:**
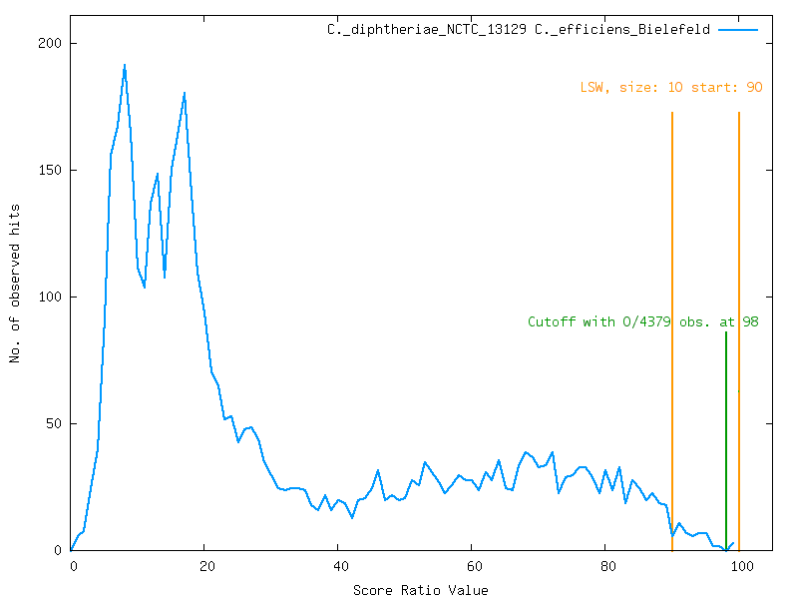
**SRVs for Corynebacterium genus**. Histogram of the SRVs (multiplied by 100 to gain percent values) resulting from the comparison of two *Corynebacterium *strains. There is no clear peak at the high score region of the histogram. The *lowest scoring window *is found at positions 90 – 100 and the lowest single value is found at 98%. In this comparison the vast majority of all BLAST hits would be left out. For this reason the cutoff for genome comparisons showing no bimodal distribution is automatically set to 35%.

In some cases the SRV distribution does not show the expected bimodal shape. This is mostly the case if there is a high variation within the genomes of a genus. A good example is the genus *Corynebacterium*, where the genomes are very diverse, leading to a SRV distribution with only one peak for low similarities and a broad plateau of medium scores with a decay at the highest scores. The cutoff calculation described above leads to cutoffs at very high SRVs, thereby omitting the majority of all BLAST observations (see Figure 2). To overcome this problem the master-cutoff is set to 35% if the majority of SRV histograms do not show a bimodal distribution. This value has shown to be a good cutoff value in the genomes compared with EDGAR.

### Data model

EDGAR stores the bidirectional best BLAST hit information of the all-against-all comparison of the genomes and all needed sequence information in a SQLite database. A modular data scheme and the project based approach allow to arbitrarily update single projects of the EDGAR database backend whenever a new genome becomes available for the respective genus. A local copy of the NCBI Bacteria database used by EDGAR is updated regularly. Based on this update newly included genomes are added to existing EDGAR projects to keep the database up-to-date.

### Calculation of the core genome and pan genome

The core genome is calculated by iterative pairwise comparison of a set of genomes *G*. One genome is selected as reference genome, the gene content of this genome is taken as base set for the following calculations. This set *A *of genes is compared to a set *B *of genes contained in another genome of the set G. For each gene in set *A*, a lookup is performed to check if it has a reciprocal best hit in set *B*. The lookup is performed on the BBHs filtered according to the orthology criterion calculated based on the SRVs. Every gene from set *A *that has no reciprocal best hit in set *B *is removed from the set. The resulting set *A' *is then iteratively compared to the genes of the remaining genomes in *G*, resulting in a final set of genes that have hits in all genomes of *G*, thus forming the core genome. The pan genome is also calculated in a similar way. A set *B *of genes is compared to the base set *A *of genes. Every gene of *B *that has no ortholog in *A *is added to the reference set. This process is repeated iteratively for all genomes in the set *G*, extending the base set *A *step by step to the pan genome. The selected reference genome has nearly no impact on the resulting core genome, its main purpose is that the genes of the reference genome appear first in the results. However, there may be some small bias (< 1%) due to paralogous genes appearing in different order during the calculation.

### Phylogenetic trees

**Figure 3:**
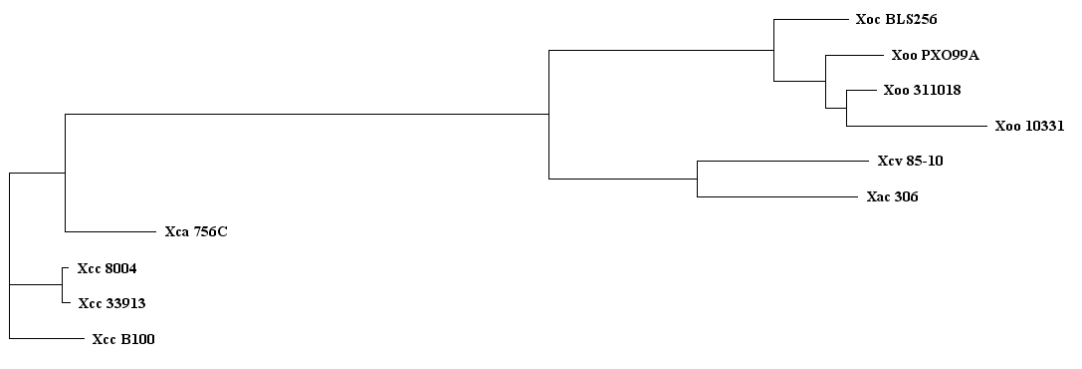
**Phylogenetic tree of Xanthomonas strains**. Phylogenetic tree of the *Xanthomonas *chromosomes currently available in public databases. Based on the core genome of 2,156 CDS the divergence of these plant-pathogenic bacteria was quantified with the recently annotated Xcc B100 employed as reference to construct the tree. The Xcc genomes and Xca cluster closely together, and are linked by a common branch to the remaining Xanthomonads. Here Xca and Xcv diverge from the *X. oryzae *chromosomes. Among these rice pathogens Xoc forms a first side branch, while the Xoo genomes cluster together.

To support the comparison of different genomes, phylogenetic trees (see Figure 3) can be generated using a slightly adapted version of the pipeline proposed by Zdobnov *et al*. [28]. The core genome is calculated as described above. In the next step multiple alignments for all core genes are created using MUSCLE [29]. Non matching parts of the alignments are masked using GBLOCKS [30] and then removed. The matching parts are concatenated to one big multiple alignment of more then 1 Mb length. Finally, a phylogenetic tree is generated from this long alignment using PHYLIP [31].

### User interface

The user interface is based on an Apache Web Server using mod_perl and CGI. The web interface is separated into three parts: The HTML code is organized in static HTML templates. These templates use XHTML 1.0 strict, which is supported by all modern browsers. The graphical layout is implemented using CSS stylesheets. Both the XHTML and the CSS code were validated to be compliant to the standards of the W3C consortium.

## Results

EDGAR is designed to support the high throughput comparison of related genomes. A comparison of the genomes of all genus groups of the NCBI genomes database with more than three sequenced strains was performed, and the resulting orthology information is made available to the scientific community.

### The EDGAR web application

EDGAR provides a precomputed database with orthology information for all genomes of a genus, based on a generic orthology criterion calculated from score ratio values (SRV – see Methods). The calculated orthologous genes as well as a number of visualization features are accessible via a full featured web interface. Genomes of identical genus are clustered together, where for each compared genus group a separate project database is created to store the BLAST score ratio based orthology information. The SRV histograms and the derived cutoffs can be plotted for every genome combination. The resulting histogram of SRV-cutoffs for all possible genome combinations is also available. Using these plots the user can validate the orthology criterion calculated by EDGAR.

Genes that have no orthologs in any other genome of the genus are called singletons. Using EDGAR the singletons of every genome in comparison to its group can be estimated within seconds. The singletons of a bacterial strain can be exported as fasta file for further analysis.

**Figure 4:**
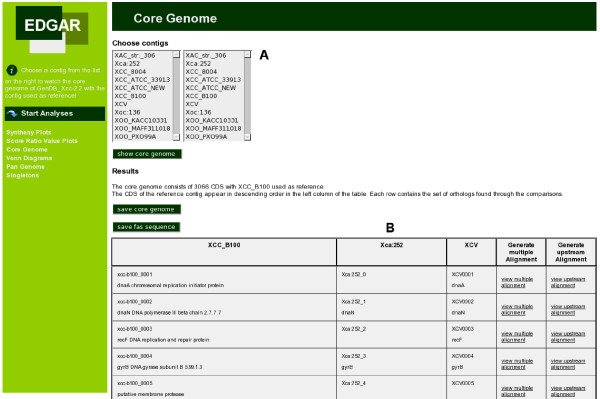
**Web interface: Core genome presentation**. Screenshot of the core genome calculation in the EDGAR web interface. In the upper part (A) one can choose a reference genome and a set of genomes to compare it with. The resulting table is shown in the lower part (B) of the page, in this case the core genome table for Xcc B100, Xca 756C, and Xcv 85-10. EDGAR displays the orthologous genes of all compared strains together with their gene function (as far as it is known) for every gene in the core genome. For every set of orthologous genes multiple alignments can be constructed of the genes itself and of their upstream region.

The core genome can be calculated for a selected reference genome in comparison to every combination of genomes of its genus. The genes of the reference genome are used as the starting set for the iterative core calculation (see Methods). The calculated core genome is presented as a table of orthologous genes of all selected genomes and their functions, starting with the selected reference genome in the first column (see Figure 4).

To observe the differences between the orthologous genes of the core genome, EDGAR features multiple alignments of the core genes, created using MUSCLE [29]. Furthermore multiple alignments are created for the upstream region of the core genes. This allows the researcher to quickly find conserved sequences in the upstream region, e.g. when searching for promotor binding sites or recognition sites of regulatory elements. The pan genome, the set of all unique genes of the compared genomes, can be calculated for user defined sets of genomes. The pan genome is also listed as a table of orthologous genes with their gene functions, beginning with all genes of the reference genome, followed by the genes added to the gene pool of the genus by the other species. The table comprises unique genes only, meaning that a gene that is orthologous to a previously listed gene will not be added to the list. The pan genome and core genome can be exported as fasta files or as TAB separated tables of locus tags.

**Figure 5:**
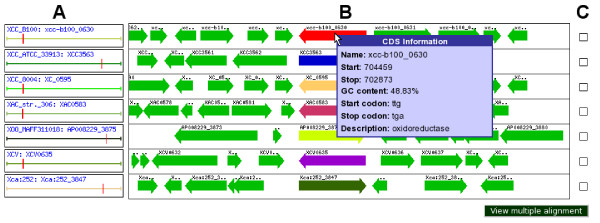
**Web interface: Comparative view**. Comparative view of seven orthologous genes of the Xanthomonas genus. In the left part (A) the location of the genes in their respective genome is shown by the red vertical marks. In the middle section (B) a linear view of the orthologous genes and their genomic neighborhood is displayed. Some information on the depicted genes can be seen by a mouseover window. The checkboxes on the right (C) allow the user to select genes for multiple alignments.

The genomic context of orthologous genes can be observed via the comparative view. The gene names in the core genome table and the pan genome table are linked to a linear view (see Figure 5) of all orthologous genes in their genomic neighborhood, which allows e.g. for the analysis of operon structures or genomic rearrangement events. The comparative view can also be accessed via the sidebar menu. Another feature of the EDGAR web interface is the creation of synteny plots. Here, stop positions of two orthologous genes of two bacterial strains are used as coordinates and plotted to a diagram with the sequence length of the compared strains serving as x/y-axis. The resulting synteny plot can reveal an insight into genome rearrangements that occurred during the evolution of the strains.

To visualize differential gene content between several genomes EDGAR can create Venn diagrams for up to five genomes. Every area in this Venn diagram represents a subset of the compared genomes and is labeled with the number of genes in this subset. To simplify the assignment of an area to a genome set every genome has a base color. The areas of the Venn diagram are colored in the averaged color of the associated genomes (based on the RGB color model). Venn diagrams for more than five genomes can be created theoretically, but as higher level diagrams get extremly complex they were not implemented in the current version of EDGAR. Projects were created for all genomes of the NCBI Bacteria database where three or more genomes of one genus were available. This resulted in 75 genus groups containing 582 genomes (as at 15.02.2009). All these projects are freely accessible via the EDGAR web interface. In order to analyze unpublished data using EDGAR, private projects with access control by a user management system can be created upon request.

### A use case study: comparative analysis of Xanthomonas genomes

In a use case study, EDGAR has been employed to compare the genomes of *Xanthomonas *strains. Xanthomonads are plant-associated and usually plant pathogenic bacteria [32]. These Gram-negative bacteria affect a broad set of host plants, among them important agricultural crops like rice and other grains [33-35], soy beans, cotton, citrus plants [36], but also tomato, pepper [37], or Crucifera [36,38] including cabbage, rape, and the model plant mouse-ear cress (*Arabidopsis thaliana*). Besides their pathogenicity-based agricultural relevance, some *Xanthomonas *species, especially *X. campestris*, are also commercially important due to their production of the polysaccharide xanthan, which found many industrial applications, mainly as a viscosifier [39-41].

Understanding the taxonomic relation of *Xanthomonas *strains has become an awkward endeavor. In the early days of microbiology, each bacterial isolate identified from a host plant for which no member of this bacterial genus had been described previously was classified as a new species [42]. Later many of these species were merged on the basis of in vitro tests, but the original name identifying the main host plant was conserved in the term "pathovar" [43]. Incorporation of information derived from partial knowledge of DNA sequences, such as 16S rDNA sequences or RFLP patterns, led then to a reassessment of the *Xanthomonas *taxonomy [44], which is still in progress [45,46]. This phylogenetic analysis provides not only the basis for a systematic order of the *Xanthomonas *bacteria, but also a deeper understanding of the evolution of the *Xanthomonas *strains. However, all attempts so far to reconstruct the true evolutionary relationships between the Xanthomonads did not lead to a taxonomy that is generally applied within the community. Instead, the differing classifications of the strains resulted in inconsistent naming in the literature. Thus, exploiting the emerging genome data may now open the door to obtain a well-established *Xanthomonas *taxonomy on a definite basis. We have used EDGAR to assess this approach.

All *Xanthomonas *genome data currently available from public sequence repositories have been employed for a comparative analysis of these bacteria. The genome data were from the *X. campestris *pv. campestris (Xcc) strains ATCC 33913 [36], 8004 [38], and B100 [39], the *X. campestris *pv. amoraciae (Xca) strain 756C, the *X. campestris *pv. vesicatoria (Xcv) strain 85-10 [37], the *X. axonopodis *pv. citri (Xac) strain 306 [36], the *X. oryzae *pv. oryzae (Xoo) strains KACC10331, [33] MAFF311018 [34], and PXO99A [35], and the *X. oryzae *pv. oryzicola (Xoc) strain BLS256. The main features of these *Xanthomonas *strains and their genomes are summarized in Table 1.

**Table 1:** Overview on Xanthomonas chromosomes

Strain	*X. campestris *pv. campestris B100	*X. campestris *pv. campestris ATCC33913	*X. campestris *pv. campestris 8004	*X. campestris *pv. amoraciae 756C	*X. campestris *pv. vesicatoria 85-10	*X. axonopodis *pv. Citri 306	*X. oryzae *pv. oryzae 10331	*X. oryzae *pv. oryzae 311018	*X. oryzae *pv. oryzae PXO99A	*X. oryzae *pv. oryzicola BLS256
Abbr^*b*^	Xcc B100	Xcc 33913	Xcc 8004	Xca 756C	Xcv 85-10	Xac 306	Xoo 10331	Xoo 311018	Xoo PXO99A	Xoc BLS256
Size(bp)	5,079,002	5,076,187	5,148,708	4,941,214	5,178,466	5,175,554	4,941,439	4,940,217	5,240,075	4,831,739
CDS	4,471	4,181	4,273	4,534	4,487	4,313	4,637	4,372	5,083	4,554
PMID^*c*^	18304669	12024217	15899963		16237009	12024217	15673718		18452608	

^*a *^This strain carries additional plasmids

^*b *^Abbreviation used in this manuscript

^*c *^Pubmed ID where available

In a first analysis, the pan genome of the *Xanthomonas *chromosomes was computed to consist of 12,951 coding sequences (CDS). Among these genes, a core genome of 2,156 CDS was determined. Besides genes encoding basic features like the central metabolism and the cell envelope, the core genome comprised genes important for survival in the bacterial environment. Such genes coded i.e. for the flagella and chemotaxis, for putative glycosidases and sugar uptake systems. Furthermore, pathogenicity factors like the type I-IV secretion systems seemed basically conserved among all so far analyzed *Xanthomonas *strains, as well as the xanthan production machinery encoded by the gum genes.

**Figure 6:**
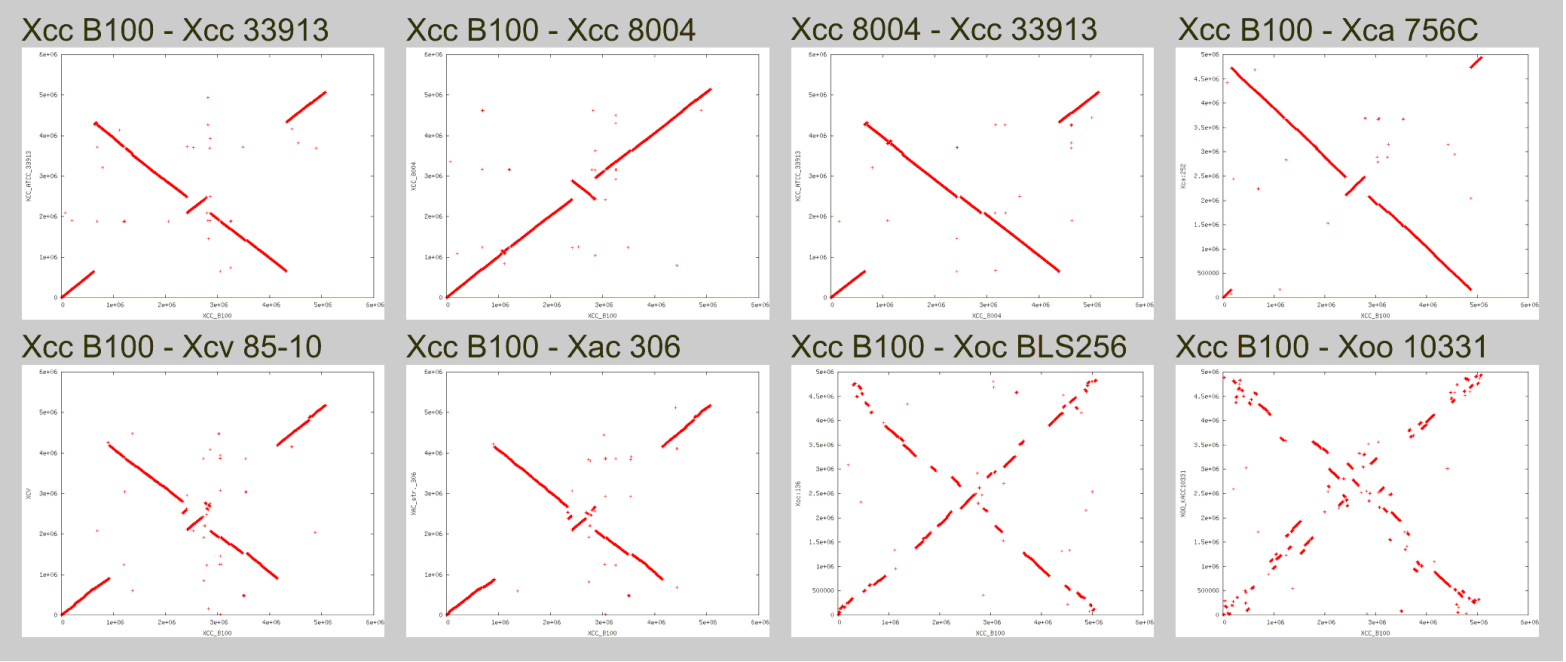
**Synteny of the Xanthomonas chromosomes**. Synteny of the *Xanthomonas *chromosomes. In order to monitor the conservation of gene order among the *Xanthomonas *chromosomes, pairwise synteny plots were generated with EDGAR, where the position of each CDS of the chromosome given on the X axis is plotted against the position of its homologue in the second chromosome given on the Y axis. Identical chromosomes result in a diagonal plot. The names of the analyzed chromosome pairs are given on top of each plot. Among the Xcc and Xca chromosomes there are few chromosomal rearrangements, some of which indicate large-scale inversion events. The number of rearrangements increases rather subtly when the Xca/Xcv chromosomes are compared to Xcc strains. A substantial increase in rearrangements becomes obvious for Xoc BLS256 when compared to Xcc B100, while the gene order seems almost disintegrated in Xoo 10331 (similar data for the other Xoo chromosomes not shown). While synteny analysis is restricted to complete genome data for obvious reasons, other tools like the phylogenetic tree analysis of the Venn diagrams are also available for draft genome data.

To get an overview on the true taxonomy of the sequenced *Xanthomonas *strains, the determined core genome data was used for a phylogenetic analysis. The genome divergence was quantified with EDGAR using a similar approach to one which recently proved to be of value for eukaryotes [28]. The result can be displayed in a tree with two main branches: the first comprising almost all *X. campestris *genomes and the second with the genomes of the *X. oryzae *strains, which includes the genomes of *X. axonopodis *and of *X. campestris *pv. vesicatoria 85-10 in a separate branch (see Figure 3). In the *X. campestris *branch, all Xcc genomes were very close to each other and the genome of Xca 756C was only marginally more distant from these strains. The second branch was more heterogeneous. Beside the branch with Xac 306 and Xcv 85-10 the genome of Xoc BLS256 was more distant from the Xoo strains. This phylogenetic clustering was in good accordance with the phytopathogenicity of the *Xanthomonas *strains; the *X. campestris *strains, which were grouped together, are all pathogens of cruciferous plants, while the *X. oryzae *strains are pathogens of rice (*Oryza sativa*). Also, Xoc BLS256, which forms a distinct branch within this group, causes bacterial leaf streak disease, while the Xoo strains provoke bacterial blight. Xcv and Xac that diverge from the second branch leading to the rice pathogens, affect pepper and citrus plants, respectively. The phylogenetic tree was derived from the core genome that comprised 2,156 CDS and thus less than half the genome of each *Xanthomonas *strain. To shed more light on the relation of the Xanthomonads, the synteny of genome pairs was plotted, which gave a rough survey on the conservation of gene order between individual strains (Figure 6). For the analysis Xcc B100 was set as a reference. The comparisons show that the gene order of Xcc B100, which despite some striking inversions was generally well conserved in the Xcc and Xca chromosomes, was increasingly disintegrated along the phylogenetic tree that indicates their divergence (Figure 4). While there was considerable conservation in the Xca/Xcv chromosomes, the number of rearrangements increased dramatically in comparison to Xoc, with the gene order almost comminuted in Xoo. Such chromosomal rearrangements have been linked to IS elements [35,38], mobile genetic elements that were found frequently in *Xanthomonas *genomes, with numbers ranging from 58 in Xcv 85-10 to 267 in Xoo PXO99A [35,37].

**Figure 7:**
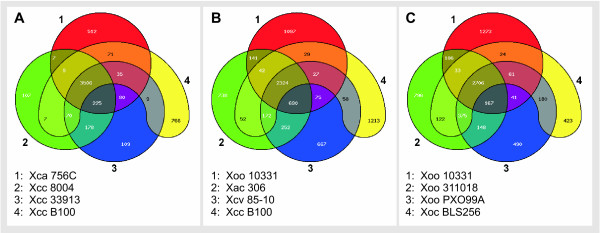
**Venn diagrams**. Venn diagrams. EDGAR facilitates visualizing common gene pools of by Venn diagrams. This analysis exploits all CDS of the genomes and is not restricted to the core genome. In each individual analysis at most 5 genomes can be included, as considering more chromosomes results in rather confusing visualization. Results for the *X. campestris *strains pathogenic to crucifers and the rice-pathogenic *X. oryzae *that were clustered in the phylogenetic analysis (Figure 4) are displayed in panels A and C, respectively. Among the *X. campestris *chromosomes in panel A a particular high similarity between Xcc 33913 and Xcc 8004 became evident. The chromosomes shared 178 orthologous CDS exclusively, and further 225 CDS conjointly with strain Xca 756C. In panel C among the *X. oryzae *genomes, the chromosomes of *X. oryzae *pv. oryzae strains shared 375 orthologs, while the *X. oryzae *pv. oryzicola chromosome overlapped less with the Xoo chromosomes. In panel B the Xac and Xcv chromosomes that clustered in between the *X. campestris *and *X. oryzae *groups were compared with each other and a representative of the *X. campestris *and *X. oryzae *groups. The analysis brought to light a surprisingly high number of 690 orthologs shared among Xac, Xcv and the Xoo representative, indicating closer connections of these strains to the *X. oryzae *group than to the crucifer pathogenic *X. campestris *strains.

The degree in gene order conservation among the *Xanthomonas *chromosomes as apparent from the synteny analysis seems well correlated with the phylogenetic order computed for the core genome CDS. Two taxonomic groups became evident, comprising of *X. campestris *strains pathogenic for crucifers and of *X. oryzae *strains pathogenic for rice. In between there was a third group consisting of Xca and Xcv 85-10. These three groups have been further characterized by analyzing the distribution of orthologous CDS within the groups (Figure 7). Among the crucifer-pathogenic *X. campestris *strains (Figure 7A) there were particular overlaps between the genomes of strain Xcc 33913 and Xcc 8004. For the genome of strain Xca 756C, that had been classified to the distinct pathovar "amoraciae", no outstanding role became obvious when compared to the Xcc strains. Among the *X. oryzae *chromosomes (Figure 7C) the Xoo strains had a large number orthologs in common, thus reflecting the different symptoms provoked by Xoc when affecting rice. The Xac/Xcv chromosomes that branched off the remaining Xanthomonads conjointly between the *X. campestris *and *X. oryzae *groups, shared many orthologs with an Xoo representative that was also included in the comparison (Figure 7B).

Altogether these analyses conveniently performed with EDGAR lead to a more comprehensive view on the phylogeny of the Xanthomonads that so far was clouded to some extend by previous contradictory taxonomic classifications. Now EDGAR facilitates a high-resolution analysis for the sequenced strains. The results for the available genomes imply two phylogenetic groups that constitute crucifer-pathogenic and rice-pathogenic strains, respectively. While the genome-based analyses reflect the distinct disease symptoms caused by an infection with *X. oryzae *pv oryzicola, the classification of Xca 756C in a separate "pathovar" is questioned. Furthermore, the *X. axonopodis *pv. citri and *X. campestris *pv. vesicatoria strains are related. This is in accordance with previous phylogenetic analyses that caused *X. campestris *pv. vesicatoria strains to be reclassified as *X. axonopodis *[44,46]. The substantial distance between Xcv 85-10 and the group of the remaining *X. campestris *strains suggest that a renaming for Xcv 85-10 should be considered.

## Discussion

As we demonstrated by the use case EDGAR provides various useful features for the comparative analysis of closely related genomes. While some of the presented features are available also in the CMR or the MBGD, EDGAR adds some novel aspects like the phylogenetic analysis or the Venn diagrams of common gene pools. The intuitive web interface and the auto-generated SRV based orthology cutoffs allow researchers to analyze genomes of their interest as quick as possible. The SRVs have been shown to be a useful method for a generic orthology threshold estimation. These generic thresholds are crucial for the high throughput comparison of genomes, as it is much too laborious to observe every genome group manually. While working well in the vast majority of cases, in some genus groups (e.g. *Corynebacterium*) the SRV cutoff calculation fails due to very dissimilar genomes. A proper method to estimate the threshold in these cases has yet to be developed, up until then a fix threshold is used. As most other orthology estimation approaches also use static thresholds, this is no major drawback.

EDGAR will be continuously enhanced, there are several features planned for the future. The identification and visualization of segmental duplications in analyzed genomes will be one of the main topics in the further development of EDGAR. Another planned feature is the integration of additional visualization features to the web interface like e.g. circular plots of orthologous genes.

A script based prototype of EDGAR was successfully used for the comparative analysis of *Neisseria meningitidis *strains. Schoen *et al*. [47] compared disease and carriage strains of *N. meningitidis *to gain insights into virulence evolution. Some techniques used in this work like a curve fitting approach to test for an "open" pan genome are also planned to be integrated into EDGAR in the near future.

Another feature to come is a search mask for boolean queries on sets of genomes. Furthermore, the integration between EDGAR and the automatic annotation framework GenDB [14] will be expanded by integrating direct links from EDGAR to GenDB annotations.

With regard to the large numbers of unfinished genomes that are expected to arise from next generation sequencing technologies, EDGAR is not limited to completely assembled genomes. As the calculation is gene based, EDGAR has the capability to analyze multiple contig draft genomes, provided that a gene finding approach like GLIMMER or CRITICA [48,49] was performed on the contigs. The comparative view of EDGAR could actually support the annotation of unfinished genomes.

Space requirements of an EDGAR project depend on the size and number of the analyzed genomes. Among the precomputed projects the space requirements vary from 9 MB (*Buchnera *– four genomes of about 500 genes) to 1.2 GB (*Mycobacterium *– 19 genomes of about 4500 genes). The compute time for one project also highly depends on the number of genes to be compared (e.g. via BLAST). Processing all 582 genomes in the precomputed projects took about three days on a compute cluster (127×Sunfire V20z dual Opteron 1.8 Ghz, 27 × SunFire X2200 dual cpu dual core Opteron 2,4 Ghz, 3 × SunFire V880 8 × Ultra Sparc III).

## Conclusion

With the rapidly emerging ultra-fast sequencing technologies the trend moves towards analyzing not just one genome, but groups of related genomes. EDGAR is the ideal tool for analyzing connatural genomes by providing a quick insight into the similarities and differences among the sequenced genomes. EDGAR was used to analyze all suitable sequences of the NCBI genomes database. All genomes were sorted by their genus, and every genus-group with three or more sequenced species was processed with EDGAR. This resulted in 75 genus groups containing a total of 582 genomes.

All these groups are accessible via the EDGAR web interface located at . Since only published genomes are used in the analysis, no access control is needed. However, it is possible to create private EDGAR projects for unpublished data upon request. The EDGAR web frontend provides convenient access to all data stored in the EDGAR databases, allowing for the fast and easy calculation of singletons, core genome, and pan genome of any combination of related genomes available in the NCBI Genomes database so far. The web based access to comparative data via EDGAR stages an ideal platform for cooperative work of researchers all over the world.

Additionally, when comparing newly sequenced genomes to a well annotated one, the orthology information applied by EDGAR can be used to transfer annotation information from the old to the new genomes. Visualization features include synteny plots for pairs of genomes, as well as Venn diagrams of up to five genomes. Phylogentic trees as presented in the use case study make a powerful expert system for evolutionary analyses available to the scientific community. The visualizations stated above as well as the singleton, core genome or pan genome tables can be easily exported for further use in other tools.

Additionally, the overview tables generated by EDGAR are the perfect means to give a review of the analyzed genomes and to identify promising genes for further inspections and specific analyses. All these features make EDGAR a valuable gain for scientists in the field of comparative genomics.

Concerning the *Xanthomonas *use case, the advancements in ultra-fast sequencing technology imply the arrival of further *Xanthomonas *genome data in the future. Easy-to-use tools like EDGAR will allow constant and timely enhancements in understanding the phylogeny of these organisms upon arrival of genome data. As increased taxon sampling greatly reduces phylogenetic errors [50], remaining obscurities in *Xanthomonas *taxonomy may be resolved efficiently by extensively exploiting genome data by means of EDGAR.

## Availability and requirements

• **Project name: **EDGAR

• **Project home page: **

• **Use case study: **project "BMC_Xanthomonas" on the EDGAR home page

• **Operating system(s): **Platform independent

• **Programming language: **Perl, JavaScript

• **Other requirements: **JavaScript enabled web browser

• **Software license: **GNU GPL

• **License agreement required for non-academic users**

## Authors' contributions

DD has designed and implemented the initial EDGAR framework. JB has supervised the prototype development and implemented the final version. FJV contributed the *Xanthomonas *use case and the phylogenetic analyses. SPA helped in the creation of the web server and in the drafting of the manuscript. MZ developed the comparative viewer. AP contributed biological background knowledge. AG supervised the development of the initial system design and approved the final manuscript. All authors have read and approved the manuscript.
